# An Attempt to Correct Erroneous Ideas Among Teacher Education Students: The Effectiveness of Refutation Texts

**DOI:** 10.3389/fpsyg.2020.577738

**Published:** 2020-10-09

**Authors:** Marta Ferrero, Emmanouil Konstantinidis, Miguel A. Vadillo

**Affiliations:** ^1^Departamento de Investigación y Psicología de la Educación, Universidad Complutense de Madrid, Madrid, Spain; ^2^Department of Psychology, University of Warwick, Coventry, United Kingdom; ^3^Departamento de Psicología Básica, Universidad Autónoma de Madrid, Madrid, Spain

**Keywords:** misconceptions, refutation texts, intervention, pre-service teachers, education

## Abstract

There is sound evidence about the high prevalence of misconceptions about education among pre-service teachers. This trend continues after students complete the degree in education and once they are in the exercise of their profession. In fact, several studies show that these misconceptions are widespread among in-service teachers. Erroneous ideas about education may divert material and human resources to poor grounded methods and teaching tools, compromising the quality of education. Strategies to debunk misconceptions among future teachers, who may not have a firm position about many educational issues, might contribute to reversing this trend. The main goal of the present study was to assess the efficacy of refutation texts in the correction of misconceptions among pre-service teachers. As in previous studies with in-service teachers, refutation texts were effective in reducing participants’ endorsement of misconceptions. But this effect was short-lived and did not affect participants’ intention to use educational methods that are based on the misconceptions addressed in the refutation texts.

## Introduction

One of the most important strategies to guarantee high quality teaching is to endow teachers with subject-matter knowledge and a repertoire of evidence-based pedagogical skills (Ingvarson and Rowe, 2008). However, teacher education students in many training colleges are often invited to rely on observation and hard-earned experience rather than on rigorous, high-quality research and evidence when selecting educational methods for the classroom (Seidenberg, 2013). Moreover, teacher education programs do not always include authoritative educational research findings (Moats, 1999; Gersten, 2001) nor content knowledge about how research is conducted and how to interpret its findings (Levin, 2013; Seidenberg, 2013; Hammersley-Fletcher and Lewin, 2015). At the same time, there is a huge market of courses, workshops, and books that offer a wide range of pseudoscientific theories and methods about how to improve learning, such as Brain Gym^®^ (Hyatt, 2007) or The Glenn Doman Method (Edkin, 1987). Not surprisingly, many pre-service teachers hold a substantial number of erroneous ideas about education. For instance, it has been shown that many of them believe that hemispheric dominance can explain individual differences among students (i.e., Fuentes and Risso, 2015; Tardif et al., 2015) or that letter reversal is a common symptom of dyslexia (i.e., Washburn et al., 2014; Soriano-Ferrer et al., 2016). This trend continues after students complete their degree in education and during the exercise of their profession. In fact, the high prevalence of erroneous ideas among in-service teachers has been widely documented all over the world (e.g., Dekker et al., 2012; Ferrero et al., 2016).

The prevalence of misconceptions among pre-service and in-service teachers can have serious consequences in the quality of education, as these beliefs pave the way for ill-grounded methodologies and might impede the adoption of effective procedures of teaching (Goswami, 2006; Busso and Pollack, 2014). To mention just a few examples, the popularity of learning styles has motivated many teachers to divert their time and resources to adapting their way of teaching to the learning styles of their students. However, there is sound evidence against this practice (Coffield et al., 2004). Similarly, the groundless idea that reading disabilities are caused by abnormal eye movements has often favored the use of optometric exercises on children with dyslexia (Handler et al., 2011), at the expense of training in well-founded aspects of literacy such as alphabetic principle or word recognition (National Reading Panel, 2000).

One possible solution to this problem is to explicitly address the erroneous ideas among teachers (Pintrich et al., 1993). Unfortunately, the available evidence shows that, once adopted, misconceptions can become quite resistant to change (Lewandowsky et al., 2012), even when they have been already recognized as erroneous by the target audience (Johnson and Seifert, 1994). In addition, not all methods to address misconceptions are equally valid and, in some cases, they can even backfire, that is, they can strengthen the target ideas instead of challenging them (i.e., Nyhan and Reifler, 2010; Nyhan et al., 2013; Nyhan and Reifler, 2015), although this finding has not always been replicated (Haglin, 2017; Swire et al., 2017; Wood and Porter, 2019).

In this context, refutation (or refutational) texts have received special attention as a simple means to change misconceptions (Guzzeti et al., 1993; Tippet, 2010; Lewandowsky et al., 2012). Refutation texts are defined as those that describe a common theory, belief, or idea, refute it, and offer a satisfactory alternative (Guzzetti, 2000). In general, the evidence collected indicates that refutation texts are a powerful tool for addressing erroneous ideas (Guzzeti et al., 1993; Tippet, 2010; Lewandowsky et al., 2012). This might be due to their effectiveness in creating some of the conditions necessary to induce a conceptual change among people. More precisely, according to Posner et al. (1982), refutation texts can provoke dissatisfaction with current conceptions and provide an alternative explanation to the audience. Preferably, this explanation must be *intelligible*, and not more difficult to understand than the current conceptions (Lombrozo, 2007); *plausible*, that is, it must be helpful to resolve the problem generated and also consistent with other knowledge; and *inspiring* to open up new areas of inquiry (Posner et al., 1982).

Refutation texts have received some attention in teacher education. For instance, Hynd et al. (1997) analyzed the changes induced by these texts in the conceptions of pre-service teachers about projectile motion. Likewise, Salisbury-Glennon and Stevens (1999) and Kutza (2000) tested the effectiveness of refutation texts to elicit a conceptual change in motivation knowledge among teacher students. Finally, Gill et al. (2004) addressed the epistemological beliefs of pre-service teachers about mathematics through the use of this tool. In Salisbury-Glennon and Stevens (1999), refutation texts were tested alone, while in the rest of studies they were assessed in combination with other elements such as real demonstrations (Hynd et al., 1997), alerts about conflicting information (Gill et al., 2004), or rewards for adjusting conceptual change to the expert opinion stated in the refutation texts (Kutza, 2000). All in all, the evidence gathered in these studies showed that refutation texts enabled the correction of erroneous ideas among teacher students although in general a full correction was not achieved and in some cases their effectiveness depended on the addition of extra elements (Kutza, 2000; Gill et al., 2004). The only study that measured the effects of refutation texts in the long run found that their impact remained significant 2 months later (Hynd et al., 1997).

In a recent experiment, we tested the use of refutation texts to correct some of the most prevalent misconceptions about education among in-service teachers (Ferrero et al., 2020). Along with this, we aimed to determine if the inclusion of information discrediting the origin of the misconception had any influence on the effectiveness of refutation texts. The results showed that, in the short run, refutation texts were effective at debunking misconceptions about education among in-service teachers, although the addition of information discrediting the origin of the misconceptions did not increase their impact. However, all the effects disappeared in a month and, most importantly, the manipulation failed to change teachers’ intention to use educational methods based on misconceptions. Overall, the results of Ferrero et al. (2020) converge with those of previous research showing that, once adopted, misconceptions are highly resistant to change.

As mentioned above, teacher education students hold a large number of misconceptions which prevail over time and might affect the exercise of their profession (Goswami, 2006; Busso and Pollack, 2014). Because of their continuous exposure to ideas and educational practices of dubious validity, in-service teachers may show positions radically opposed to the message presented in refutation texts. In contrast, pre-service teachers may not yet have firm positions on several educational issues and hence an intervention focused on debunking misconceptions in this sample might yield more promising results. Following up on this hypothesis, the aim of the present study was to replicate the results of Ferrero et al. (2020) with teacher education students. In brief, the experiment consisted of three phases. During Phase 1, we measured the prevalence of different misconceptions through a multiple-choice questionnaire. During Phase 2, we exposed each participant to three conditions (refutation text with information about the origin of the misconception, refutation text alone, and no text) and immediately afterward we measured again the prevalence of the target misconceptions. During Phase 3, we measured for a last time the prevalence of the misconceptions. We introduced two modifications in comparison with Ferrero et al. (2020). First since the degree of endorsement for misconceptions had no effect in the preceding experiments with in-service teachers, we did not consider this variable in the analyses. Second, as the timing of the present study coincided with participants’ completion of their undergraduate degree, we included two additional questions aimed at exploring whether participants had received or searched for extra information about the target misconceptions over the course of the experiment.

## Materials and Methods

### Participants

As in Ferrero et al. (2020), to recruit participants for the study, we sent personal invitations to the headmaster of each college by email. After accepting to participate, we jointly established the schedule of the research. The day before the start of each phase, the first author (MF) sent the link of the corresponding experimental task to the teachers who agreed to collaborate in the experiment. On the intervention days, students received the link from their teachers and completed the tasks during class time.

Due to the difficulty in recruiting the target sample, our intention was to test the maximum number of participants that we could reach using the same recruitment strategy as in Ferrero et al. (2020). The power analysis conducted in Ferrero et al. (2020, Experiment 2) shows that at least 23 participants are needed to detect an effect of the manipulation on misconceptions in Phase 2 in a two-tailed test with 85% power. The final sample included 64 elementary education majors (40 female) from two different education colleges in the Basque Country, Spain. The mean age of the sample was 20.47 (*SD* = 1.52). Participants were enrolled in the second (42%) and third year (58%) of the college degree.

### Materials

Unless noted otherwise, the materials were identical to those of Experiment 2 in Ferrero et al. (2020). All these materials are available in the Supplementary Material.

#### Phase 1

We employed a three-part questionnaire. The first part contained an informed-consent form and requested background information about the participants. The second part contained 36 statements about education and neuroscience applied to education. Eighteen of them hold well-grounded evidence and the remaining half are based on null or very weak evidence and can be considered misconceptions. Participants were asked to judge the validity of each statement using a 5-point Likert scale ranging from 1 (Definitively false) to 5 (Definitively true). Although the questionnaire assessed endorsement for 36 statements, only nine of them were addressed in the experimental manipulation described below (Phase 2). Responses to the remaining 27 items were ignored in the statistical analyses. The third part of the questionnaire included 18 educational interventions. Half of these approaches referred to well-grounded practices, while the remaining nine referred to practices with very poor or null evidence that corresponded to the nine target misconceptions addressed during the intervention. Participants were asked to rate their intention to use or recommend each methodological approach through a 6-point Likert scale ranging from 1 (Definitively not) to 6 (Definitively yes). Only responses to the nine interventions addressed in the experimental manipulation (described below) were considered in the statistical analyses.

#### Phase 2

During this phase, nine of the 18 misconceptions included in the 36-item questionnaire were addressed (for the selection of the nine target misconceptions, see Ferrero et al., 2020). For each misconception, there was one refutation text with three different versions: (a) refutation text with an explanation of the origin of information and its credibility (TO); (b) refutation text alone (TA); (c) no text (NT). All the texts followed the same structured: At the beginning, the target misconception was introduced and, immediately afterward, it was refuted. Next, the origin of misinformation was discredited (only in the text-and-origin condition). Then, the alternative (and correct) information was presented. Finally, a rhetorical question was formulated.

#### Phase 3

As in Experiment 2 in Ferrero et al. (2020), during this phase, we employed the same questionnaire of Phase 1 with three additional questions. In the first two questions, students had to report whether they had searched or received additional information about the nine target misconceptions during the participation in the study. For each misconception, there were four response options: (1) I have not searched for information; (2) I do not remember having searched for information; (3) I have searched for information and it runs in the same direction of the refutation text; (4) I have searched for information and it runs in the opposite direction of the refutation text. To assess whether participants had received any information regarding each misconception during the study, in the second question “search for” was replaced by “received.” The third question was aimed at measuring the level of difficulty of the refutation texts as perceived by students. To this aim, there was a Likert scale which ranged from 1 (Extremely easy) to 10 (Extremely difficult).

### Design and Procedure

We conducted a within-subject study which consisted of three phases. During Phase 1, participants completed the on-line questionnaire described in the “Materials” section. Average completion time for Phase 1 was approximately 15 min.

As explained above, during Phase 2, nine misconceptions were assigned to three types of refutation texts described above (TO, TA, and NT). Consequently, each participant read six refutation texts in total (3 TO and 3 TA). Texts were presented in a random order for each student. Students could read each text as many times as they wished. Immediately after reading the texts, participants completed the same questionnaire used in Phase 1 for a second time. Average completion time for these two tasks (reading the texts and completing the questionnaire) was approximately 25 min. Between Phase 1 and Phase 2 there was a delay of 6 to 7 weeks.

During Phase 3, 30 days after Phase 2, participants completed the same questionnaire used in Phase 1 and Phase 2 for a third time along with the three additional questions described in the “Materials” section.

Participants completed the three phases of the study in a computer room of their college within the usual schedule. All the sessions were supervised by a teacher. The materials were presented on-line.

## Results

**FIGURE 1:**
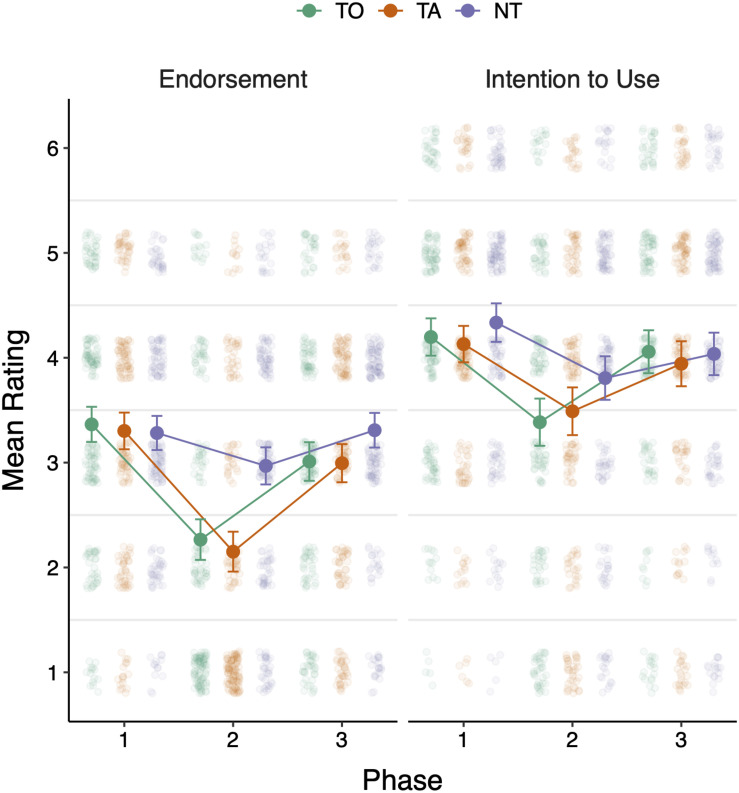
Participants’ endorsement (**left** panel) and intention-to-use ratings (**right** panel) on Phases 1–3. TO, TA, and NT refer to Text + Origin, Text Alone, and No Text, respectively. Error bars denote 95% CIs.

Figure 1 (left panel) plots the average endorsement ratings for the nine target misconceptions across experimental Conditions and Phases. The first observation that stands out is that utilizing refutational texts seems to have an effect on the rate of statement endorsement, as there is a decrease from Phase 1 to Phase 2. However, this effect does not seem to be long-lasting as there is an increase in endorsement rates in Phase 3.

We analyzed the data using a linear mixed-effects model with Condition (within-subjects; three levels: TO, TA, and NT) and Phase (within-subjects; three levels: Phases 1, 2, and 3) as fixed effects, and participant-specific random intercepts. The analysis showed a significant interaction between Condition and Phase, *χ^2^*(4) = 28.60, *p <* 0.001, suggesting that timing of intervention is crucial for the actual effect of intervention: specifically, as expected in Phase 1 there is no difference between conditions (all *post hoc* pairwise comparisons *ps* > 0.50). In Phase 2, the effect of the refutational texts causes a decrease in the rate of endorsement in the TO and TA conditions (both significantly different from the NT condition, *ps* < 0.001), but there is no difference between these two conditions, *t*(1672) = 0.11, *p* = 0.63. The effect of the refutation text (TO and TA conditions) diminishes in Phase 3 as there is an increase in the endorsement rates compared to Phase 2 (which reaches the endorsement ratings before any intervention is provided - as in Phase 1). While ratings in TO and TA conditions are still significantly different than the NT condition (both *p*s < 0.04), the present results suggest that temporal proximity to the actual intervention is an important determinant of the effectiveness of such interventions. Also, the main effects of Condition, *χ^2^*(2) = 30.08, *p <* 0.001, and Phase, *χ^2^*(2) = 152.95, *p <* 0.001, were significant.

We conducted the same type of analysis for the intention-to-use scale. Figure 1 (right panel) presents a similar picture as with endorsement ratings, that is, a decrease in the intention-to-use ratings in Phase 2 followed by an increase in Phase 3. Overall, we observed a significant main effect of Phase, *χ^2^*(2) = 72.76, *p <* 0.001, suggesting that the intention to use was lowest in Phase 2 and highest in Phase 1. Similarly, the main effect of Condition was significant, *χ^2^*(2) = 8.17, *p* = 0.017: this is driven by a significant difference between TO and NT, *t*(1669) = 2.62, *p* = 0.02, whereas the remaining two pairwise differences (TA vs NT and TA vs TO) were not significant (both *ps* > 0.05). The interaction did not reach significance, *χ^2^*(4) = 5.36, *p* = 0.252. In addition, we included the judged difficulty as a covariate in both mixed-effects models (endorsement and intention-to-use ratings), but it did not result in better predictive power (likelihood ratio tests, both *ps* > 0.45).

We also explored whether searching or receiving information about the presented statements had an effect on the endorsement and intention-to-use ratings. For this analysis, we treated the four response options (see section “Materials and Methods,” *Phase 3*) as a categorical predictor in a linear mixed-effects model which also included Condition (and their interaction) as a fixed effect and participant-specific random intercepts. This is a rather exploratory piece of analysis as the independent variable is created based on participants’ responses and it is not the result of usual methods of experimentation (i.e., random allocation to conditions). For example, in the search behavior question, the majority of participants had searched for additional information, with categories 3 and 4 accounting for 80% of all responses. For the endorsement ratings, we found an effect of search behavior, *χ^2^*(3) = 13.01, *p* = 0.005. Following up the significant main effect with pairwise *post hoc* tests (Tukey’s adjustments), the only difference was observed between the extreme responses 1 (“I have not searched for information”; *M*_*R1*_ = 3.33) and 3 [“I have searched for information and it runs in the same direction of the refutation text”; *M*_*R3*_ = 2.87; *t*(499) = 3.24, *p* = 0.007]. This result suggests that searching for information which is consistent with the refutation text can potentially decrease endorsement ratings for inaccurate statements. This effect does not seem to be moderated by condition as both the main effect and the interaction did not reach significance (both *ps* > 0.25). The results about the intention-to-use ratings are similar: the main effect of search was significant, *χ^2^*(3) = 16.85, *p* < 0.001: as in the endorsement ratings, those statements that left unexplored received highest usage ratings (*M*_*R1*_ = 4.38) as opposed to those statements that were searched for and for which the information found was in line with the refutation text [*M*_*R3*_ = 3.86; *t*(586) = 2.83, *p* = 0.025]. There were also significant differences between response categories 1 and 2 [*M*_*R*2_ = 3.39; *t*(560) = 3.77, *p* = 0.01], and 2 and 4 [*M*_*R*4_ = 4.12; *t*(552) = 2.66, *p* = 0.041].

The pattern of results when considering whether participants had received any information about the misconceptions was similar to that of search behavior. The responses for this question are more balanced than the search behavior (R1 = 30.08%; R2 = 9.22%; R3 = 35.13%; R4 = 25.57%). In terms of endorsement ratings, the main effect of receiving information was significant, *χ^2^*(3) = 25.46, *p* < 0.001, with reliable pairwise differences between response category 1 (*M*_*R1*_ = 3.22) and 3 [*M*_*R3*_ = 2.64; *t*(501) = 4.74, *p* < 0.001], and 3 and 4 [*M*_*R4*_ = 3.03; *t*(375) = 3.15, *p* = 0.0097]. The interaction with condition was not significant, *χ^2^*(6) = 4.64, *p* = 0.59. For the intention-to-use ratings, we observed the same pattern: the main effect of receiving information is significant, *χ^2^*(3) = 46.17, *p* < 0.001, with reliable pairwise differences between response category 1 (*M*_*R1*_ = 4.35) and 3 [*M*_*R3*_ = 3.59; *t*(587) = 6.69, *p* < 0.001], 3 and 4 [*M*_*R4*_ = 3.98; *t*(573) = 3.65, *p* = 0.0017], and 1 and 4, *t*(569) = 2.62, *p* = 0.044.

## Discussion

The high prevalence of misconceptions about education among teacher education students is well-documented (i.e., Tardif et al., 2015; Soriano-Ferrer et al., 2016). These erroneous ideas, which are usually not corrected during college years and are even promoted through different channels, might jeopardize the adoption of effective methods in the classroom. Despite this, until now only a handful of studies have directly tried to combat this type of ideas (Im et al., 2018; Ferrero et al., 2020). The aim of the present study was to replicate the research of Ferrero et al. (2020) in a sample of pre-service teachers.

The results showed that refutation texts might reduce the number of misconceptions among teacher education students. Specifically, when presented within a refutation text, participants significantly reduced their belief in those erroneous ideas in comparison with the beliefs that were not refuted. As in Ferrero et al. (2020), adding information about the origin of the misconceptions (TO) did not produce better results than not providing it (TA). Once again, this result runs in the opposite direction of some studies which found that undermining the reliability of the misinformation or its source might promote beliefs correction (Lewandowsky et al., 2005; Guillory and Geraci, 2013). Interestingly, we found that the effect of refutation texts did not last over time. Thirty days after the intervention, the effects of refutation texts had decreased significantly. These results are in perfect agreement with the study performed with in-service teachers (Ferrero et al., 2020) and suggest that the effectiveness of refutation texts is largely determined by temporal proximity to the intervention. The reason that could explain the differences between these results and those obtained in the study of Hynd et al. (1997), where long-term effects were found, may lie in the type of ideas that were discredited in each case. In the latter, the misconceptions were about physics. Unlike educational topics, natural phenomena can inspire more confidence in expert voices and, in turn, not be so dependent on a community’s cultural heritage.

Along with the reduction on the number of misconceptions, we were also interested in measuring the impact of refutation texts on the reduction of participants’ intention to use educational practices that were based on the misconceptions refuted in the texts. Our results do not lend support to the hypothesis that the refutation texts changed participants’ willingness to adopt educational practices that were based on the misconceptions. Although, intention-to-use ratings were numerically lower in the two conditions with refutation texts (TO and TA) than in the control condition (NT), these differences were already present in Phase 1, although not significant. And, in any case, there is no evidence whatsoever that those differences persisted in Phase 3. These results are also in line with our previous experiment with in-service teachers.

In the present study, we also explored whether after reading the refutation texts participants searched or were presented with additional information about the target misconceptions. About 80% of them stated that they searched actively for information and 61% stated that they had received information about the misconceptions. In general, those who searched or received information challenging the misconception showed lower endorsement and intention-to-use ratings than participants who did not search or receive this information or received information supporting the misconception. These results confirm that students receive a substantial amount of information about these misconceptions in their field of education (Moats, 1999; Gersten, 2001) and that this information does not always challenge the myth. In our analyses, whether or not students encountered information for or against, each misconception did not interact with the experimental manipulation. But it did have a main effect on endorsement ratings and intention-to-use ratings. Participants who actively searched for information and found that it run in the same direction as the refutation text showed, overall, lower endorsement and intention-to use ratings than participants who did not search for information. And participants who (passively) received information in agreement with the refutation text gave lower endorsement and intention-to-use ratings than those who did not receive any information at all or received information supporting the misconception. This fact is not trivial because teachers prefer known and nearby sources (Landrum et al., 2002; Cook and Schirmer, 2003) and, therefore, the rigor of the information sources closest to the centers play a crucial role. These findings have been confirmed in the present study, where challenging information found by students have had an effect on their beliefs.

Regardless of domain knowledge, misconceptions have been proven to be extremely resistant to change (Lewandowsky et al., 2012). In fact, individuals persist in relying on them even when they can recall a correction (Johnson and Seifert, 1994). Faced with this, some researchers have suggested that efforts to correct misinformation should target only to people with moderate rather that strong beliefs (Ecker et al., 2014). To some extent, the results of the present experiment support this recommendation. Participants in the study reduced their belief in misconceptions after reading the refutation text, but this effect disappeared shortly after the intervention and did not change their intention to use practices based on the refuted misconceptions. Future research should explore alternative means to extend the effects of refutation texts in the long run both on beliefs and educational practices. In this regard, it would be interesting to test the efficacy of refutation texts combined with other strategies that may maximize their impact, such as discussion groups, training in the scientific method, or inoculation. The latter is proving to be a promising strategy in several disciplines such as health or politics (Banas and Rains, 2010) and might be a welcome option to correct misconceptions among pre- and in-service teachers. For instance, this technique could be tested by warning participants that they are about to be fooled by incorrect information. In the same line, it would be valuable to measure the effects of refutation texts, alone or accompanied by other strategies, at different intervals to determine which is the most effective formula to get a verifiable and permanent impact on educational ideas and practices among pre- and in-service teachers.

## Data Availability Statement

The raw data supporting the conclusions of this article will be made available by the authors, without undue reservation.

## Ethics Statement

This study was reviewed by the King’s College London Ethics Committee. The participants provided their written informed consent to participate in this study.

## Author Contributions

All authors listed have made a substantial, direct and intellectual contribution to the work, and approved it for publication.

## Conflict of Interest

The authors declare that the research was conducted in the absence of any commercial or financial relationships that could be construed as a potential conflict of interest.
