# Supplementary material for: An Attempt to Correct Erroneous Ideas Among Teacher Education Students: The Effectiveness of Refutation Texts
Source: Front Psychol. 2020 Oct 9;11:577738. doi: 10.3389/fpsyg.2020.577738 (PMC7581673; doi:10.3389/fpsyg.2020.577738)
Supplement: Supplementary file 1 [file Table_1.DOCX]

Table 1. *Items with robust evidence.*

| 1. Direct instruction leads to better outcomes than discovery learning. |
| --- |
| 2. Reversing letters is NOT a symptom of dyslexia. |
| 3. The impact of new technologies on learning is questionable. |
| 4. Boys have bigger brains than girls. |
| 5. It is more effective to give feedback that includes information about the correct answer than information about the incorrect answer. |
| 6. Accelerated learning is an appropriate approach for gifted students. |
| 7. The proportion of boys diagnosed with autism is greater than the proportion of girls. |
| 8. Normal development of the human brain involves the birth and death of brain cells. |
| 9. Extended rehearsal of some mental processes can change the shape and structure of some parts of the brain. |
| 10. Phonological knowledge, alphabetic principle, fluency, vocabulary, and comprehension are key elements in the teaching of reading. |
| 11. The left and right hemispheres of the brain always work together. |
| 12. Production of new connections in the brain can continue into old age. |
| 13. Homework is more beneficial for secondary students than for elementary students. |
| 14. We use our brains 24 h a day. |
| 15. Spacing practice is more effective than concentrating the same amount of practice in a shorter period of time. |
| 16. Differences between boys and girls are negligible when teaching one another. |
| 17. Information is stored in the brain in a network of cells distributed throughout the brain. |
| 18. To diagnose a child with autism, symptoms should be present in two or more contexts (i.e. at home and at school). |

Table 2. *Items with null or very weak evidence.*

| 1. Environments that are rich in stimulus improve the brains of pre-school children. (*) | |
| --- | --- |
| 2. Individuals learn better when they receive information in their preferred learning style (e.g., auditory, visual, and kinesthetic). (*) |  |
| 3. Adapting teaching methods to the “multiple intelligences” of students leads to better learning. | |
| 4. Exercises that rehearse co-ordination of motor-perception skills can improve literacy skills. (*) | |
| 5. Short bouts of coordination exercises can improve integration of left and right hemispheric brain function. | |
| 6. Children with learning difficulties and autism can benefit from controlled sessions of sensorial stimulation (i.e., being swayed in an hammock or be stroked with a brush). | |
| 7. Differences in hemispheric dominance (left brain, right brain) can help explain individual differences among learners. (*) | |
| 8. Several reading difficulties can be remediated by optometric exercises (i.e., to follow the movement of a ball with the eyes, to number letters line by line using exclusively the eyes). | |
| 9. New generations of students have sophisticated technical skills that helps them to learn more efficiently using information from the Internet. (*) | |
| 10. We only use 10% of our brain. (*) | |
| 11. Learning and behaviour disorders can be reduced by short bouts of listening to electronically modified music (i.e., Berard method). | |
| 12. The number of children with autism has increased significantly over the last years. | |
| 13. Listening to the classical music of Mozart enhances childrens’ intelligence. (*) | |
| 14. Crossed laterality (i.e., right hand dominant and left eye dominant) is associated with learning disabilities. (*) | |
| 15. The whole-language approach (teaching whole words and not the correspondence letter-sound) is most appropriate in the early teaching of reading. (*) | |
| 16. There are critical periods in childhood after which certain things can no longer be learned. | |
| 17. The measles-mumps-rubella vaccine might cause autism. | |
| 18. The majority of babies (3-18 months) can learn to read using an appropriate method. | |

(*) Misconceptions addressed in Phase 2.

Spanish versions of the nine refutation texts used during Phase 2 of the study and an English approximate translation of them are shown below. Sentences in bold contain the explanation added in the TS condition about the source of information and its credibility.

| **Text 1**  There is a common belief that environments that are rich in stimuli improve the development of neuronal connections that enhance intelligence in pre-school children. However, this belief is incorrect. **It is based on the results of rats reared in extreme sensorial deprivation, far removed from the conditions under which a child grows up.** Neurologists show that, except for extreme cases, the formation of connections occurs outside of environmental stimulation. On the one hand, experts show that the formation of neuronal connections begins even before birth, this is, before children receive stimulation from the environment. On the other hand, many studies have found that, in normal conditions, stimuli from the environment do not cause a large formation of neuronal connections even after birth. Moreover, who says that having more neuronal connections (synapses) is better? Several disorders demonstrate that having particularly a high number of synapses is not desirable. People with Fragile-X syndrome is a clear example.  **Texto 1**  Existe la creencia popular de que los entornos ricos en estímulos incrementan las conexiones cerebrales y por tanto la inteligencia de los niños pre-escolares. Sin embargo, esta creencia es errónea. **Se basa en los resultados con ratas criadas en condiciones de deprivación sensorial extrema, muy alejadas de las condiciones en las que crece un niño humano.** Los neurólogos demuestran que, salvo en casos extremos, la formación de conexiones en el cerebro ocurre al margen de la estimulación ambiental. Por un lado, los expertos muestran que la formación de conexiones neuronales comienza incluso antes del nacimiento, es decir, antes de que los bebés reciban ninguna estimulación del ambiente. Por otro lado, muchos estudios encuentran que, en condiciones normales, después del nacimiento los estímulos ambientales tampoco provocan una mayor formación de conexiones neuronales. Más aún, ¿quién ha dicho que tener más conexiones neuronales (sinapsis) sea mejor? Muchos trastornos evidencian que tener niveles de sinapsis especialmente altos no es deseable. Un claro ejemplo de esto son las personas con el síndrome de X-frágil. |
| --- |
| **Text 2**  The belief that individuals learn better when they receive information in their preferred learning style (e.g., auditory, visual, and kinesthetic) has become very popular. However, it must be noted that this is false and is considered a myth by OECD. **It arose in 1975 when a psychologist popularized a classification of students according to four learning styles, simply on the basis of his personal experience and not on empirical studies.** None of the numerous reviews performed have found that adapting teaching to the preferred learning style benefits students. In addition, classifying a student within a unique style is not always possible and the available tools to do so lack reliability and validity. This does not mean that the same type of instruction is effective in all contexts, subjects and students. But there is a significant gap between this general idea and deciding what type of instruction a student needs based on the commercialised programmes inspired by learning styles. Moreover, on the assumption that the theory is valid, would it be possible to personalize instruction for all students taking into account that there are more than 71 classifications of learning styles on the market?  **Texto 2**  La creencia de que las personas aprenden mejor cuando reciben la información en su estilo de aprendizaje preferido se ha hecho muy popular. Ahora bien, conviene decir que es falsa. **Se originó en 1975 cuando un psicólogo popularizó una clasificación de los estudiantes en función de cuatro estilos de aprendizaje basándose simplemente en su experiencia personal y no en estudios empíricos.** Ninguna de las numerosas revisiones realizadas encuentra que adaptar la enseñanza al estilo de aprendizaje preferido beneficie a los alumnos. Además no siempre es posible clasificar a un estudiante dentro de un solo estilo y las herramientas disponibles para hacerlo carecen de fiabilidad y validez. Esto no significa que el mismo tipo de instrucción valga en cualquier contexto y materia y para todos los alumnos. Pero de ahí a supeditar la decisión de qué tipo de instrucción necesita cada alumno a los comercializados estilos de aprendizaje hay una brecha importante. Incluso, suponiendo que la teoría fuera válida, ¿sería posible personalizar la enseñanza para todos los alumnos considerando que existen más de 71 clasificaciones de estilos de aprendizaje en el mercado? |
| **Text 3**  Programs that promote perceptuo-motor coordination exercises to improve literacy skills have become very popular. However, these programs are ineffective. **They originate from well-known commercial products such as Brain Gym® or Primitive reflexes inhibition therapy whose authors, who are making great fortunes, have been strongly discredited by education and health authorities.** These programs state that doing specific physical exercises can modify the structure of the brain and improve the acquisition of reading, among others. However, cognitive psychology has shown that improving perceptuo-motor skills does not cause the improvement of other higher-order processes such as reading. If this were so, wouldn’t it be difficult to find children with dyslexia who are very good at sports? Undoubtedly, this is not the case. Psycholinguistics have shown that complex skills such as reading only improve through direct and specific training. Thus, reading programs that focus on phonological knowledge, grapheme-phoneme correspondence, fluency, vocabulary and comprehension have been shown to be effective at improving reading in several empirical studies.  **Texto 3**  Los programas que promueven hacer ejercicios de coordinación perceptivo-motora para mejorar la lecto-escritura se han hecho muy populares. Sin embargo, estos programas son ineficaces. **Su origen está en conocidos productos comerciales como el Brain Gym® o la terapia de inhibición de reflejos primitivos cuyos autores, que están amasando grandes fortunas, han sido desacreditados duramente por las autoridades educativas y sanitarias.** Estos programas defienden que realizar determinados ejercicios físicos puede modificar la estructura del cerebro y mejorar así el aprendizaje lector, entre otros. Sin embargo, la psicología cognitiva muestra que mejorar las habilidades perceptivo-motoras no causa la mejora de otros procesos superiores como la lectura. Si así fuera, ¿no debería ser muy difícil encontrar niños disléxicos a los que se les dé muy bien el deporte? Sin duda, no es el caso. Los psicolingüistas evidencian que las habilidades complejas como la lectura sólo mejoran si se entrenan de forma directa y específica. Así, los programas que trabajan el conocimiento fonológico, la unión letra-sonido, la fluidez lectora, el vocabulario y la comprensión sí han mostrado una gran eficacia en la mejora de la lectura en numerosos estudios científicos. |
| **Text 4**  There is a common belief that we only use 10% of our brain. However, it should be noted that this is false. **This belief originates from a misinterpretation by the first researchers to study the brain. At that time and with limited resources, they overlooked the function of 90% of this organ.** Nowadays, clinic neurology has shown that if we only used 10% of our brain, we would be in a vegetative state. None of the brain areas can be destroyed without provoking some kind of functional impairment. In fact, losing much less than 90% of our brain as a result of an accident or an illness has already catastrophic consequences for people, such as a loss of speech or memory. In addition, neurophysiological techniques have shown that there is no area in the brain which is never used. Finally, in light of evolutionary theory, would it make sense that our metabolism spent so many resources in a greatly underutilized organ?  **Texto 4**  La creencia de que sólo usamos el 10% de nuestro cerebro es muy popular en los medios. Sin embargo, conviene subrayar que es falsa. **Todo comenzó tras una interpretación equivocada de las declaraciones de los primeros investigadores en el cerebro. Éstos, por aquel entonces y con escasos medios, afirmaron que aún desconocían la función del 90% de este órgano.** Hoy en día, la neurología clínica demuestra que si sólo usáramos el 10% de nuestro cerebro estaríamos en estado vegetativo. Ninguna área del cerebro puede ser destruida sin dejar en la persona algún tipo de daño funcional. De hecho, la pérdida de áreas muy pequeñas del cerebro a causa de un accidente o de una enfermedad ya tiene consecuencias catastróficas en las personas, tales como la pérdida del habla o la memoria. Las técnicas de exploración neurofisiológica, a su vez, demuestran que no hay ninguna parte del cerebro que no se use nunca. Por último, bajo la lógica de la evolución, ¿tendría algún sentido que nuestro metabolismo hiciera un gasto tan elevado de recursos en un órgano tan infrautilizado? |
| **Text 5**  Over the past few years, the belief that differences in hemispheric dominance (left brain, right brain) can help explain individual differences among learners has become popular. However, this idea is incorrect. **It arose from overgeneralization to healthy people of results obtained with epileptic patients who underwent removal of their corpus callosum, a brain structure that unites the two cerebral hemispheres.** At present, brain scans made with neuroimaging techniques show that both hemispheres work together during all cognitive activities. It is true that there is some hemispheric specialisation for specific skills but, at the same time, in a normal brain there are a large number of inter-hemispheric connections. Moreover, there is no evidence that differences among people in terms of creativity, logic or capacity to get excited are reliably linked to processing differences in either hemisphere. In other words, did you know that all people, from the most creative ones to the most analytic ones, employed the two hemispheres continuously?  **Texto 5**  Desde hace unos años, se ha extendido la idea de que los estudiantes, y las personas en general, pueden ser clasificados como "de cerebro derecho" (persona artística, creativa, emocional) o "de cerebro izquierdo" (persona racional, analítica, lógica). No obstante, esta idea es errónea. **Nació a partir de la sobregeneralización a población sana de los resultados obtenidos con pacientes epilépticos a los que se extirpaba el cuerpo calloso que une los dos hemisferios cerebrales para estudiar sus reacciones.** Actualmente, las exploraciones del cerebro hechas mediante técnicas de neuroimagen muestran que ambos hemisferios trabajan juntos en todas las tareas cognitivas. Sí es cierto que existe cierta especialización hemisférica para determinadas habilidades pero, al mismo tiempo, en un cerebro normal hay una grandísima cantidad de conexiones inter-hemisféricas. Es más, no hay evidencia que demuestre que las diferencias entre personas en términos de creatividad, lógica o capacidad de emocionarse estén relacionadas con diferencias de procesamiento de uno u otro hemisferio. Dicho de otra forma, ¿sabía que todas las personas, desde las más creativas hasta las más analíticas, utilizan ambos hemisferios continuamente? |
| **Text 6**  There is a common belief that new generations of students have sophisticated technological skills that enable them to learn more efficiently using information on the web. Nevertheless, this belief is incorrect. **It emerged when in 2011 an advisor and designer of videogames coined the term “digital native” to describe the youth of today on the basis of anecdotes and informal observations.** Both objective indicators and information obtained though structured interviews with young people of the “new digital age” show that they do not have a better knowledge about how to use technologies to improve their knowledge than previous generations. They utilize identical technologies as other age groups, such as mobile phones. And they rarely utilize learning tools, such as blogs. In addition, they have trouble building knowledge from on-line information, as shown by the incorrect use of search keywords or by a fleeting stay on web pages that precludes an appropriate evaluation. Finally, did you know that most students would prefer to use fewer technological tools in the classroom?  **Texto 6**  Existe la creencia popular de que las nuevas generaciones de estudiantes poseen habilidades tecnológicas sofisticadas para construir nuevos aprendizajes a partir de información de la web. No obstante, esta creencia es incorrecta. **Surgió cuando en 2001 un asesor y diseñador de videojuegos acuñó el término "nativo digital" para describir a los jóvenes de hoy en día basándose en anécdotas y observaciones informales.** Tanto los indicadores objetivos como la información recogida mediante entrevistas estructuradas a jóvenes de la "nueva era digital" muestran que éstos no tienen un conocimiento mayor de cómo utilizar las tecnologías para mejorar su aprendizaje. Usan tecnologías idénticas a las que usan el resto de grupos, como los móviles, y apenas emplean herramientas de creación de aprendizaje, como los blogs. Además, tienen dificultad para construir conocimiento a partir de información on-line, como muestra el empleo incorrecto de las palabras clave de búsqueda o una permanencia fugaz en las páginas web que impide su correcta evaluación. Por último, ¿sabía que la mayoría de los estudiantes universitarios preferiría que no se usaran tanto las tecnologías en el aula? |
| **Text 7**  The belief that listening to the classical music of Mozart increases the intelligence of children is very popular. However, it is now well known to be false. **It arose from a controversial and misrepresented paper published in *Nature* which reported that the spatial intelligence of undergraduate students increased after listening to the classical music of Mozart.** To begin with, the results obtained in assessing the effects of music of Mozart were not conclusive. While some studies obtained improvements, others did not obtain any effect. In addition, in several of these studies, the sample consisted of adults and not of children. Furthermore, in the few cases where improvements were obtained, they occurred in spatial-temporal reasoning, never in general intelligence. Improvements also varied depending on the type of spatial tasks chosen. Moreover when these improvements occurred, they did not last forever but only for a few minutes. Finally, isn’t it suspicious that the greatest promoters of Mozart effect are companies and websites of products for children that are making profits of millions of euros?  **Texto 7**  La creencia de que escuchar música clásica, especialmente de Mozart, aumenta la inteligencia de los niños es muy popular. Sin embargo, hoy en día se sabe que es falsa. **Surgió de un estudio controvertido y tergiversado de la revista Nature donde se encontraba que la inteligencia espacial de un grupo de universitarios mejoraba tras escuchar música de Mozart.** Para empezar, los resultados obtenidos al evaluar los efectos de la música de Mozart no son concluyentes. Mientras algunos estudios científicos encuentran mejoras, otros no. Además, muchos de estos estudios se han hecho con adultos y no con niños. Por otra parte, las pocas investigaciones que han encontrado mejoras, lo hacen únicamente en el razonamiento espacio-temporal, nunca en la inteligencia general. Y además varían en función del tipo de tareas espaciales escogidas. Más aún, estas mejoras, cuando las hay, no se mantienen para siempre sino que apenas duran unos minutos tras escuchar la música. Por último, ¿no es muy sospechoso que los mayores impulsores del efecto Mozart sean empresas y webs de productos infantiles que están haciendo un negocio de millones de euros gracias a él? |
| **Text 8**  Several teachers believe that crossed laterality (i.e., right dominant hand and left dominant eye) is associated with learning difficulties. However, this belief is incorrect. **The origin of this belief lies in an intervention method that was created in 1960 to impose "hemispheric dominance" and that has been strongly criticized and censored by the scientific and medical community.** Numerous studies have attempted to verify if there is any association between crossed laterality and academic achievement, but practically none of them has found any result that confirms this link. Most studies clearly show that crossed laterality is not a predictor of lower intelligence in children. Nor is it a sign of more inaccurate or slower reading. Similarly, crossed laterality does not predict spelling difficulties. Nor is it associated with arithmetic difficulties. These results are constant across different age groups. Finally, if crossed laterality is so serious, is it not suspicious that there are no evidence-based interventions to address it yet?  **Texto 8**  Muchos maestros creen que la lateralidad cruzada (p.ej., mano dominante derecha y ojo dominante izquierdo) es un factor asociado a las dificultades de aprendizaje. Sin embargo, esta idea es incorrecta. **El origen de esta creencia está en un método de intervención que se creó en 1960 para "imponer la dominancia hemisférica" y que ha sido duramente criticado y censurado por la comunidad científica y médica.** Numerosos estudios han intentado comprobar si hay relación entre la lateralidad cruzada y el rendimiento académico, pero prácticamente ninguno ha encontrado resultados que confirmen esta asociación. La gran mayoría de investigaciones muestra de forma sólida que el hecho de que un niño tenga lateralidad cruzada no es predictor de una menor inteligencia. Tampoco lo es de una lectura más lenta o imprecisa. Asimismo, la lateralidad cruzada no predice las dificultades en escritura. Y tampoco está asociada a dificultades en aritmética. Estos resultados se aplican a niños de cualquier edad. Por último, si tan grave es, ¿no resulta sospechoso que aún no exista ni un solo tratamiento para corregir la lateralidad cruzada con evidencia científica? |
| **Text 9**  The idea that the global method (to teach whole words instead of grapheme-phoneme correspondences) is the most appropriate for reading instruction is very popular. However, it is erroneous. **The idea arose when the global method was imported from Anglo-Saxon countries, ignoring that the phonology of English is completely different from ours.** In Spanish, Catalonian, Basque or Galician each sound always corresponds to the same letter, with very few exceptions. If we teach a child these correspondences, they will be able to read independently and accurately any word. English irregularities have fostered the use of the global method, but even Anglo-Saxon countries are returning to the use of grapheme-phoneme correspondences. The numerous studies performed over the last 20 years clearly show the superiority of the synthetic method over the global method to acquire a fluent and accurate reading ability, specifically in children with learning difficulties. In addition, cognitive psychologists have found no evidence that the global method improves reading ability. Finally, did you know that learning the name or the sound of letters is one of the most important predictors of reading success in the future?  **Texto 9**  La idea de que el método global (enseñar a leer palabras completas y no la asociación letra-sonido) es el más adecuado para la enseñanza de la lectura es muy popular. Ahora bien, esta idea es errónea. **Se inició con la importación de esta metodología desde países anglosajones, sin considerar que la fonología del inglés es muy diferente a la nuestra.** En castellano, catalán, euskera o gallego a cada letra le corresponde siempre un mismo sonido, salvo contadas excepciones. Si a un niño le enseñamos estas correspondencias, podrá leer solo y sin errores cualquier palabra. Las irregularidades del inglés han favorecido el uso del método global pero incluso en los países anglosajones se está retornando a la correspondencia letra-sonido. Los numerosos estudios de los últimos 20 años muestran de forma robusta la superioridad del método sintético sobre el método global para adquirir una lectura fluida y precisa, especialmente en niños con dificultades. Además, desde la psicología cognitiva tampoco hay evidencia de que el método global facilite un aprendizaje lector más significativo. Por último, ¿sabía que aprender el nombre de las letras o su sonido es uno de los principales predictores del éxito lector futuro? |
