# Supplementary material for: An Attempt to Correct Erroneous Ideas Among Teacher Education Students: The Effectiveness of Refutation Texts
Source: Front Psychol. 2020 Oct 9;11:577738. doi: 10.3389/fpsyg.2020.577738 (PMC7581673; doi:10.3389/fpsyg.2020.577738)
Supplement: Supplementary file 3 [file Data_Sheet_2.PDF]

NEUROCIENCIA Y EDUCACIÓN

La finalidad de las lecturas que se van a presentar a continuación es determinar cuál es el modo más eficaz de transmitir conocimientos sobre neurociencia y educación a profesorado en activo o en formación. El tiempo estimado para leer los textos es de 10 minutos.

Después de las lecturas, aparece un breve cuestionario. El tiempo estimado para responder a las preguntas del mismo es de 10 minutos.

Antes de comenzar, asegúrese de que cuenta con el tiempo suficiente para completar las dos tareas.

La participación en el estudio es voluntaria. Usted puede abandonarlo en cualquier momento. El cuestionario es anónimo y los datos serán tratados de manera estadística y confidencial. Si desea conocer en mayor detalle los códigos éticos que rigen nuestro trabajo como psicólogos e investigadores, puede consultar el portal del Consejo General de la Psicología de España: www.cop.es

*Obligatorio

1. Introduzca los cuatro últimos dígitos y la letra de su DNI (p. ej., 2503c) *

   Le pedimos este dato simplemente para ligar sus respuestas de la Fase 2 con las de la Fase 1 y 3 y, a la vez, garantizar su anonimato.

2. A continuación, se presentan seis textos expositivos breves sobre neurociencia y educación. Por favor, lea atentamente cada uno de ellos. *

   Selecciona todos los que correspondan.

   ☐ De acuerdo

3. Existe la creencia popular de que los entornos ricos en estímulos incrementan las conexiones cerebrales y por tanto la inteligencia de los niños pre-escolares. Sin embargo, esta creencia es errónea. SE BASA EN LOS RESULTADOS CON RATAS CRIADAS EN CONDICIONES DE DEPRIVACIÓN SENSORIAL EXTREMA, MUY ALEJADAS DE LAS CONDICIONES EN LAS QUE CRECE UN NIÑO HUMANO. Los neurólogos demuestran que, salvo en casos extremos, la formación de conexiones en el cerebro ocurre al margen de la estimulación ambiental. Por un lado, los expertos muestran que la formación de conexiones neuronales comienza incluso antes del nacimiento, es decir, antes de que los bebés reciban ninguna estimulación del ambiente. Por otro lado, muchos estudios encuentran que, en condiciones normales, después del nacimiento los estímulos ambientales tampoco provocan una mayor formación de conexiones neuronales. Más aún, ¿quién ha dicho que tener más conexiones neuronales (sinapsis) sea mejor? Muchos trastornos evidencian que tener niveles de sinapsis especialmente altos no es deseable. Un claro ejemplo de esto son las personas con el síndrome de X-frágil. *

Selecciona todos los que correspondan.

- [ ] He leído y comprendido el texto.

4. La creencia de que sólo usamos el 10% de nuestro cerebro es muy popular en los medios. Sin embargo, conviene subrayar que es falsa. TODO COMENZÓ TRAS UNA INTERPRETACIÓN EQUIVOCADA DE LAS DECLARACIONES DE LOS PRIMEROS INVESTIGADORES EN EL CEREBRO. ÉSTOS, POR AQUEL ENTONCES Y CON ESCASOS MEDIOS, AFIRMARON QUE AÚN DESCONOCÍAN LA FUNCIÓN DEL 90% DE ESTE ÓRGANO. Hoy en día, la neurología clínica demuestra que si sólo usáramos el 10% de nuestro cerebro estaríamos en estado vegetativo. Ninguna área del cerebro puede ser destruida sin dejar en la persona algún tipo de daño funcional. De hecho, la pérdida de áreas muy pequeñas del cerebro a causa de un accidente o de una enfermedad ya tiene consecuencias catastróficas en las personas, tales como la pérdida del habla o la memoria. Las técnicas de exploración neurofisiológica, a su vez, demuestran que no hay ninguna parte del cerebro que no se use nunca. Por último, bajo la lógica de la evolución, ¿tendría algún sentido que nuestro metabolismo hiciera un gasto tan elevado de recursos en un órgano tan infrautilizado? *

Selecciona todos los que correspondan.

- [ ] He leído y comprendido el texto.

5. La creencia de que escuchar música clásica, especialmente de Mozart, aumenta la inteligencia de los niños es muy popular. Sin embargo, hoy en día se sabe que es falsa. SURGIÓ DE UN ESTUDIO CONTROVERTIDO Y TERGIVERSADO DE LA REVISTA NATURE DONDE SE ENCONTRABA QUE LA INTELIGENCIA ESPACIAL DE UN GRUPO DE UNIVERSITARIOS MEJORABA TRAS ESCUCHAR MÚSICA DE MOZART. Para empezar, los resultados obtenidos al evaluar los efectos de la música de Mozart no son concluyentes. Mientras algunos estudios científicos encuentran mejoras, otros no. Además, muchos de estos estudios se han hecho con adultos y no con niños. Por otra parte, las pocas investigaciones que han encontrado mejoras, lo hacen únicamente en el razonamiento espacio-temporal, nunca en la inteligencia general. Y además varían en función del tipo de tareas espaciales escogidas. Más aún, estas mejoras, cuando las hay, no se mantienen para siempre sino que apenas duran unos minutos tras escuchar la música. Por último, ¿no es muy sospechoso que los mayores impulsores del efecto Mozart sean empresas y webs de productos infantiles que están haciendo un negocio de millones de euros gracias a él? *

Selecciona todos los que correspondan.

- [ ] He leído y comprendido el texto.

6. La creencia de que las personas aprenden mejor cuando reciben la información en su estilo de aprendizaje preferido se ha hecho muy popular. Ahora bien, conviene decir que es falsa. Ninguna de las numerosas revisiones realizadas encuentra que adaptar la enseñanza al estilo de aprendizaje preferido beneficie a los alumnos. Además no siempre es posible clasificar a un estudiante dentro de un solo estilo y las herramientas disponibles para hacerlo carecen de fiabilidad y validez. Esto no significa que el mismo tipo de instrucción valga en cualquier contexto y materia y para todos los alumnos. Pero de ahí a supeditar la decisión de qué tipo de instrucción necesita cada alumno a los comercializados estilos de aprendizaje hay una brecha importante. Incluso, suponiendo que la teoría fuera válida, ¿sería posible personalizar la enseñanza para todos los alumnos considerando que existen más de 71 clasificaciones de estilos de aprendizaje en el mercado? *

Selecciona todos los que correspondan.

- [ ] He leído y comprendido el texto.

7. Desde hace unos años, se ha extendido la idea de que los estudiantes, y las personas en general, pueden ser clasificados como "de cerebro derecho" (persona artística, creativa, emocional) o "de cerebro izquierdo" (persona racional, analítica, lógica). No obstante, esta idea es errónea. Actualmente, las exploraciones del cerebro hechas mediante técnicas de neuroimagen muestran cómo ambos hemisferios trabajan juntos en todas las tareas cognitivas. Sí es cierto que existe cierta especialización hemisférica para determinadas habilidades pero, al mismo tiempo, en un cerebro normal hay una grandísima cantidad de conexiones inter-hemisféricas. Es más, no hay evidencia que demuestre que las diferencias entre personas en términos de creatividad, lógica o capacidad de emocionarse estén relacionadas con diferencias de procesamiento de uno u otro hemisferio. Dicho de otra forma, ¿sabía que todas las personas, desde las más creativas hasta las más analíticas, utilizan ambos hemisferios continuamente? *

Selecciona todos los que correspondan.

- [ ] He leído y comprendido el texto.

8. Muchos maestros creen que la lateralidad cruzada (p.ej., mano dominante derecha y ojo dominante izquierdo) es un factor asociado a las dificultades de aprendizaje. Sin embargo, esta idea es incorrecta. Numerosos estudios han intentado comprobar si hay relación entre la lateralidad cruzada y el rendimiento académico, pero prácticamente ninguno ha encontrado resultados que confirmen esta asociación. La gran mayoría de investigaciones muestra de forma sólida que el hecho de que un niño tenga lateralidad cruzada no es predictor de una menor inteligencia. Tampoco lo es de una lectura más lenta o imprecisa. Asimismo, la lateralidad cruzada no predice las dificultades en escritura. Y tampoco está asociada a dificultades en aritmética. Estos resultados se aplican a niños de cualquier edad. Por último, si tan grave es, ¿no resulta sospechoso que aún no exista ni un solo tratamiento para corregir la lateralidad cruzada con evidencia científica? *

Selecciona todos los que correspondan.

- [ ] He leído y comprendido el texto.

CUESTIONARIO

A continuación se presentan una serie de afirmaciones. Por favor, para cada cuestión marque la opción que mejor represente su valoración.

Las opciones son las siguientes:

1. Seguro que es falso

2. Creo que es falso

3. No sabe / No contesta

4. Creo que es verdadero

5. Seguro que es verdadero

Por favor, responda a las preguntas de forma individual

9. *

Selecciona todos los que correspondan.

☐ De acuerdo

10. Los niños con dificultades de aprendizaje y autismo pueden beneficiarse de sesiones controladas de estimulación sensorial (p.ej., ser balanceado en una hamaca o frotado con un pincel). *

Marca solo un óvalo.

	1	2	3	4	5	
Seguro que es falso	◯	◯	◯	◯	◯	Seguro que es verdadero

11. La mayoría de los bebés (3-18 meses) pueden aprender a leer con un método de enseñanza adecuado. *

Marca solo un óvalo.

	1	2	3	4	5	
Seguro que es falso	◯	◯	◯	◯	◯	Seguro que es verdadero

12. El conocimiento fonológico, el principio alfabético, la fluidez, el vocabulario y la comprensión son pilares básicos en la enseñanza de la lectura. *

Marca solo un óvalo.

	1	2	3	4	5	
Seguro que es falso	⬭	⬭	⬭	⬭	⬭	Seguro que es verdadero

13. El método global (enseñar a leer palabras completas y no la asociación letra-sonido) es el más adecuado para la enseñanza inicial de la lectura. *

Marca solo un óvalo.

	1	2	3	4	5	
Seguro que es falso	⬭	⬭	⬭	⬭	⬭	Seguro que es verdadero

14. Hay períodos críticos en la infancia después de los cuales ciertas cosas ya no pueden ser aprendidas. *

Marca solo un óvalo.

	1	2	3	4	5	
Seguro que es falso	⬭	⬭	⬭	⬭	⬭	Seguro que es verdadero

15. Los métodos de enseñanza que se adaptan a las inteligencias múltiples de los estudiantes conducen a un mejor aprendizaje. *

Marca solo un óvalo.

	1	2	3	4	5	
Seguro que es falso	⬭	⬭	⬭	⬭	⬭	Seguro que es verdadero

16. El impacto que tienen las nuevas tecnologías en el aprendizaje es cuestionable. *

Marca solo un óvalo.

	1	2	3	4	5	
Seguro que es falso	◯	◯	◯	◯	◯	Seguro que es verdadero

17. Las nuevas generaciones de estudiantes poseen habilidades tecnológicas sofisticadas para construir nuevos aprendizajes a partir de información de la web. *

Marca solo un óvalo.

	1	2	3	4	5	
Seguro que es falso	◯	◯	◯	◯	◯	Seguro que es verdadero

18. Para diagnosticar a un niño con TDAH, los síntomas se tienen que presentar en dos o más entornos (p.ej., en casa y en la escuela). *

Marca solo un óvalo.

	1	2	3	4	5	
Seguro que es falso	◯	◯	◯	◯	◯	Seguro que es verdadero

19. La lateralidad cruzada (p.ej., mano dominante derecha y ojo dominante izquierdo) es un factor asociado a las dificultades de aprendizaje. *

Marca solo un óvalo.

	1	2	3	4	5	
Seguro que es falso	◯	◯	◯	◯	◯	Seguro que es verdadero

20. El aprendizaje acelerado es una medida adecuada para los niños con altas capacidades. *

Marca solo un óvalo.

	1	2	3	4	5	
Seguro que es falso	◯	◯	◯	◯	◯	Seguro que es verdadero

21. Los casos de niños y niñas con autismo han aumentado significativamente durante los últimos años. *

Marca solo un óvalo.

	1	2	3	4	5	
Seguro que es falso	◯	◯	◯	◯	◯	Seguro que es verdadero

22. Las diferencias en el hemisferio dominante (cerebro izquierdo, cerebro derecho) pueden ayudar a explicar las diferencias individuales entre estudiantes. *

Marca solo un óvalo.

	1	2	3	4	5	
Seguro que es falso	◯	◯	◯	◯	◯	Seguro que es verdadero

23. Espaciar en el tiempo la práctica de lo aprendido es más efectivo que concentrar esa misma cantidad de práctica en un espacio de tiempo más corto. *

Marca solo un óvalo.

	1	2	3	4	5	
Seguro que es falso	◯	◯	◯	◯	◯	Seguro que es verdadero

24. Sesiones cortas de ejercicios de coordinación pueden mejorar la integración de la función cerebral del hemisferio izquierdo y derecho. *

Marca solo un óvalo.

	1	2	3	4	5	
Seguro que es falso	◯	◯	◯	◯	◯	Seguro que es verdadero

25. La vacuna triple vírica puede provocar autismo. *

Marca solo un óvalo.

	1	2	3	4	5	
Seguro que es falso	◯	◯	◯	◯	◯	Seguro que es verdadero

26. Los ejercicios que promueven la coordinación de las habilidades perceptivo-motoras pueden mejorar las destrezas en lecto-escritura. *

Marca solo un óvalo.

	1	2	3	4	5	
Seguro que es falso	◯	◯	◯	◯	◯	Seguro que es verdadero

27. La práctica repetida de algunos procesos mentales puede cambiar la forma y la estructura de algunas partes del cerebro. *

Marca solo un óvalo.

	1	2	3	4	5	
Seguro que es falso	◯	◯	◯	◯	◯	Seguro que es verdadero

28. Los niños tienen el cerebro más grande que las niñas. *

Marca solo un óvalo.

	1	2	3	4	5	
Seguro que es falso						Seguro que es verdadero

29. Las diferencias entre los niños y las niñas son irrelevantes a la hora de enseñar a unos y a otros. *

Marca solo un óvalo.

	1	2	3	4	5	
Seguro que es falso						Seguro que es verdadero

30. Las personas aprenden mejor cuando reciben la información en su estilo de aprendizaje preferido (p.ej., auditivo, visual y cinestésico). *

Marca solo un óvalo.

	1	2	3	4	5	
Seguro que es falso						Seguro que es verdadero

31. Los hemisferios izquierdo y derecho del cerebro siempre trabajan juntos. *

Marca solo un óvalo.

	1	2	3	4	5	
Seguro que es falso						Seguro que es verdadero

32. Los deberes tienen mayores beneficios en alumnos de secundaria que en alumnos de primaria. *

Marca solo un óvalo.

	1	2	3	4	5	
Seguro que es falso						Seguro que es verdadero

33. Muchos problemas en lectura se pueden solucionar mediante ejercicios optométricos (p.ej., seguir los movimiento de una pelota con los ojos, contar letras línea por línea usando sólo los ojos). *

Marca solo un óvalo.

	1	2	3	4	5	
Seguro que es falso						Seguro que es verdadero

34. El desarrollo normal del cerebro humano implica el nacimiento y muerte de células cerebrales. *

Marca solo un óvalo.

	1	2	3	4	5	
Seguro que es falso						Seguro que es verdadero

35. Es más eficaz dar feedback incluyendo información sobre la respuesta correcta que incluyendo información sobre la respuesta incorrecta. *

Marca solo un óvalo.

	1	2	3	4	5	
Seguro que es falso						Seguro que es verdadero

36. La información se almacena en una red de células distribuidas por todo el cerebro. *

Marca solo un óvalo.

	1	2	3	4	5	
Seguro que es falso	◯	◯	◯	◯	◯	Seguro que es verdadero

37. Solo usamos el 10% del cerebro. *

Marca solo un óvalo.

	1	2	3	4	5	
Seguro que es falso	◯	◯	◯	◯	◯	Seguro que es verdadero

38. La producción de nuevas conexiones en el cerebro puede continuar hasta la vejez. *

Marca solo un óvalo.

	1	2	3	4	5	
Seguro que es falso	◯	◯	◯	◯	◯	Seguro que es verdadero

39. Usamos el cerebro 24 horas al día. *

Marca solo un óvalo.

	1	2	3	4	5	
Seguro que es falso	◯	◯	◯	◯	◯	Seguro que es verdadero

40. Se pueden reducir los problemas de aprendizaje y conducta mediante sesiones cortas de escucha de música modificada electrónicamente (p.ej., método Berard). *

Marca solo un óvalo.

	1	2	3	4	5	
Seguro que es falso	◯	◯	◯	◯	◯	Seguro que es verdadero

41. Los entornos que son ricos en estímulos mejoran los cerebros de los niños y niñas preescolares. *

Marca solo un óvalo.

	1	2	3	4	5	
Seguro que es falso	◯	◯	◯	◯	◯	Seguro que es verdadero

42. La instrucción directa (programación previa de los contenidos y de los criterios de evaluación, secuenciación de la dificultad, feedback, modelado, práctica guiada) conduce a mejores resultados que el aprendizaje por descubrimiento (p.ej., aprendizaje por proyectos, aprendizaje basado en problemas). *

Marca solo un óvalo.

	1	2	3	4	5	
Seguro que es falso	◯	◯	◯	◯	◯	Seguro que es verdadero

43. Escuchar música de Mozart aumenta la inteligencia de los niños. *

Marca solo un óvalo.

	1	2	3	4	5	
Seguro que es falso	◯	◯	◯	◯	◯	Seguro que es verdadero

44. Escribir letras en espejo NO es un síntoma de la dislexia. *

Marca solo un óvalo.

	1	2	3	4	5	
Seguro que es falso						Seguro que es verdadero

45. La proporción de niños diagnosticados con autismo es superior a la de las niñas. *

Marca solo un óvalo.

	1	2	3	4	5	
Seguro que es falso						Seguro que es verdadero

¿Usaría o recomendaría el uso de alguna de las siguientes prácticas educativas?
Por favor, para cada cuestión marque la opción que mejor represente su valoración.

LEA ATENTAMENTE las opciones disponibles:

1. Seguro que NO
2. Muy improbable
3. Improbable
4. Probable
5. Muy probable
6. Seguro que SÍ

46. *

Selecciona todos los que correspondan.

- [ ] De acuerdo

47. Enseñar habilidades de autorregulación como facilitadoras del aprendizaje (p.ej., estrategias de organización y planificación). *

Marca solo un óvalo.

	1	2	3	4	5	6	
Seguro que NO	◯	◯	◯	◯	◯	◯	Seguro que SÍ

48. Métodos para promover el aprendizaje autónomo de los estudiantes a través de la web. *

Marca solo un óvalo.

	1	2	3	4	5	6	
Seguro que NO	◯	◯	◯	◯	◯	◯	Seguro que SÍ

49. Adaptar la enseñanza a los estilos de aprendizaje. *

Marca solo un óvalo.

	1	2	3	4	5	6	
Seguro que NO	◯	◯	◯	◯	◯	◯	Seguro que SÍ

50. Proveer al alumnado de feedback sobre qué ha hecho y cómo puede mejorar. *

Marca solo un óvalo.

	1	2	3	4	5	6	
Seguro que NO	◯	◯	◯	◯	◯	◯	Seguro que SÍ

51. Práctica distribuida de lo aprendido (espaciar en el tiempo los episodios de aprendizaje de un determinado contenido). *

Marca solo un óvalo.

	1	2	3	4	5	6	
Seguro que NO	⬭	⬭	⬭	⬭	⬭	⬭	Seguro que SÍ

52. Instrucción directa (programación previa de los contenidos y de los criterios de evaluación, secuenciación de la dificultad, feedback, modelado, práctica guiada). *

Marca solo un óvalo.

	1	2	3	4	5	6	
Seguro que NO	⬭	⬭	⬭	⬭	⬭	⬭	Seguro que SÍ

53. La práctica de determinados ejercicios físicos para restablecer o consolidar la lateralidad en casos de niños con lateralidad cruzada. *

Marca solo un óvalo.

	1	2	3	4	5	6	
Seguro que NO	⬭	⬭	⬭	⬭	⬭	⬭	Seguro que SÍ

54. Métodos de estimulación temprana para mejorar el cerebro de los niños y niñas preescolares (p.ej., Doman). *

Marca solo un óvalo.

	1	2	3	4	5	6	
Seguro que NO	⬭	⬭	⬭	⬭	⬭	⬭	Seguro que SÍ

55. Proveer al alumnado de ejemplos de problemas ya resueltos paso a paso. *

Marca solo un óvalo.

	1	2	3	4	5	6	
Seguro que NO	◯	◯	◯	◯	◯	◯	Seguro que SÍ

56. Evaluación frecuente de lo aprendido (como parte o no de la calificación final). *

Marca solo un óvalo.

	1	2	3	4	5	6	
Seguro que NO	◯	◯	◯	◯	◯	◯	Seguro que SÍ

57. Métodos para favorecer el uso del 100% del cerebro. *

Marca solo un óvalo.

	1	2	3	4	5	6	
Seguro que NO	◯	◯	◯	◯	◯	◯	Seguro que SÍ

58. Uso del método fonológico o sintético para la enseñanza inicial de la lectura (enseñar de forma explícita la asociación letra-sonido). *

Marca solo un óvalo.

	1	2	3	4	5	6	
Seguro que NO	◯	◯	◯	◯	◯	◯	Seguro que SÍ

59. Escuchar música de Mozart para aumentar la inteligencia. *

Marca solo un óvalo.

	1	2	3	4	5	6	
Seguro que NO	◯	◯	◯	◯	◯	◯	Seguro que SÍ

60. Ejercicios de coordinación para mejorar la integración de la función cerebral del hemisferio izquierdo y derecho. *

Marca solo un óvalo.

	1	2	3	4	5	6	
Seguro que NO	◯	◯	◯	◯	◯	◯	Seguro que SÍ

61. Método global para la enseñanza de la lectura (enseñar a leer palabras completas y no la asociación letra-sonido). *

Marca solo un óvalo.

	1	2	3	4	5	6	
Seguro que NO	◯	◯	◯	◯	◯	◯	Seguro que SÍ

62. Aprendizaje cooperativo (versus aprendizaje individual). *

Marca solo un óvalo.

	1	2	3	4	5	6	
Seguro que NO	◯	◯	◯	◯	◯	◯	Seguro que SÍ

63. Aceleración de curso en alumnos de altas capacidades. *

Marca solo un óvalo.

	1	2	3	4	5	6	
Seguro que NO	◯	◯	◯	◯	◯	◯	Seguro que SÍ

64. Ejercicios de coordinación de las habilidades perceptivo-motoras para favorecer la lecto-escritura. *

Marca solo un óvalo.

	1	2	3	4	5	6	
Seguro que NO	◯	◯	◯	◯	◯	◯	Seguro que SÍ

65. Puntúe del 1 (muy fácil) al 10 (muy difícil) la dificultad de los textos que acaba de leer. *

Marca solo un óvalo.

	1	2	3	4	5	6	7	8	9	10	
Muy fácil	◯	◯	◯	◯	◯	◯	◯	◯	◯	◯	Muy difícil

Este contenido no ha sido creado ni aprobado por Google.

Google Formularios
