# Supplementary material for: An Attempt to Correct Erroneous Ideas Among Teacher Education Students: The Effectiveness of Refutation Texts
Source: Front Psychol. 2020 Oct 9;11:577738. doi: 10.3389/fpsyg.2020.577738 (PMC7581673; doi:10.3389/fpsyg.2020.577738)
Supplement: Supplementary file 4 [file Data_Sheet_3.PDF]

NEUROCIENCIA Y EDUCACIÓN

A continuación se presenta un cuestionario sobre educación y neurociencia aplicada a la educación. La finalidad de este cuestionario es recoger qué conocimientos tiene el profesorado en activo o en formación sobre estas materias. El tiempo estimado para responder a las preguntas es de 15 minutos.

La participación en el estudio es voluntaria. Usted puede abandonarlo en cualquier momento. El cuestionario es anónimo y los datos serán tratados de manera estadística y confidencial. Si desea conocer en mayor detalle los códigos éticos que rigen nuestro trabajo como psicólogos e investigadores, puede consultar el portal del Consejo General de la Psicología de España: www.cop.es

*Obligatorio

Sección sin título

1. *

Selecciona todos los que correspondan.

☐ He leído y entendido las condiciones del estudio y deseo participar en el mismo de forma voluntaria.

2. Introduzca los cuatro últimos dígitos y la letra de su DNI (p. ej., 2503c) *

Le pedimos este dato simplemente para ligar sus respuestas de la Fase 3 con las de la Fase 1 y 2 y, a la vez, garantizar su anonimato.

____

CUESTIONARIO

A continuación se presentan una serie de afirmaciones. Por favor, para cada cuestión marque la opción que mejor represente su valoración.

Las opciones son las siguientes:

1. Seguro que es falso

2. Creo que es falso

3. No sabe / No contesta

4. Creo que es verdadero

5. Seguro que es verdadero

Por favor, responda a las preguntas de forma individual

3. *

Selecciona todos los que correspondan.

- [ ] De acuerdo

4. Los niños con dificultades de aprendizaje y autismo pueden beneficiarse de sesiones controladas de estimulación sensorial (p.ej., ser balanceado en una hamaca o frotado con un pincel). *

Marca solo un óvalo.

	1	2	3	4	5	
Seguro que es falso	◯	◯	◯	◯	◯	Seguro que es verdadero

5. La mayoría de los bebés (3-18 meses) pueden aprender a leer con un método de enseñanza adecuado. *

Marca solo un óvalo.

	1	2	3	4	5	
Seguro que es falso	◯	◯	◯	◯	◯	Seguro que es verdadero

6. El conocimiento fonológico, el principio alfabético, la fluidez, el vocabulario y la comprensión son pilares básicos en la enseñanza de la lectura. *

Marca solo un óvalo.

	1	2	3	4	5	
Seguro que es falso	◯	◯	◯	◯	◯	Seguro que es verdadero

7. El método global (enseñar a leer palabras completas y no la asociación letra-sonido) es el más adecuado para la enseñanza inicial de la lectura. *

Marca solo un óvalo.

	1	2	3	4	5	
Seguro que es falso	◯	◯	◯	◯	◯	Seguro que es verdadero

8. Hay períodos críticos en la infancia después de los cuales ciertas cosas ya no pueden ser aprendidas. *

Marca solo un óvalo.

	1	2	3	4	5	
Seguro que es falso	◯	◯	◯	◯	◯	Seguro que es verdadero

9. Los métodos de enseñanza que se adaptan a las inteligencias múltiples de los estudiantes conducen a un mejor aprendizaje. *

Marca solo un óvalo.

	1	2	3	4	5	
Seguro que es falso	◯	◯	◯	◯	◯	Seguro que es verdadero

10. El impacto que tienen las nuevas tecnologías en el aprendizaje es cuestionable. *

Marca solo un óvalo.

	1	2	3	4	5	
Seguro que es falso	◯	◯	◯	◯	◯	Seguro que es verdadero

11. Las nuevas generaciones de estudiantes poseen habilidades tecnológicas sofisticadas para construir nuevos aprendizajes a partir de información de la web. *

Marca solo un óvalo.

	1	2	3	4	5	
Seguro que es falso						Seguro que es verdadero

12. Para diagnosticar a un niño con TDAH, los síntomas se tienen que presentar en dos o más entornos (p.ej., en casa y en la escuela). *

Marca solo un óvalo.

	1	2	3	4	5	
Seguro que es falso						Seguro que es verdadero

13. La lateralidad cruzada (p.ej., mano dominante derecha y ojo dominante izquierdo) es un factor asociado a las dificultades de aprendizaje. *

Marca solo un óvalo.

	1	2	3	4	5	
Seguro que es falso						Seguro que es verdadero

14. El aprendizaje acelerado es una medida adecuada para los niños con altas capacidades. *

Marca solo un óvalo.

	1	2	3	4	5	
Seguro que es falso						Seguro que es verdadero

15. Los casos de niños y niñas con autismo han aumentado significativamente durante los últimos años. *

Marca solo un óvalo.

	1	2	3	4	5	
Seguro que es falso						Seguro que es verdadero

16. Las diferencias en el hemisferio dominante (cerebro izquierdo, cerebro derecho) pueden ayudar a explicar las diferencias individuales entre estudiantes. *

Marca solo un óvalo.

	1	2	3	4	5	
Seguro que es falso						Seguro que es verdadero

17. Espaciar en el tiempo la práctica de lo aprendido es más efectivo que concentrar esa misma cantidad de práctica en un espacio de tiempo más corto. *

Marca solo un óvalo.

	1	2	3	4	5	
Seguro que es falso						Seguro que es verdadero

18. Sesiones cortas de ejercicios de coordinación pueden mejorar la integración de la función cerebral del hemisferio izquierdo y derecho. *

Marca solo un óvalo.

	1	2	3	4	5	
Seguro que es falso						Seguro que es verdadero

19. La vacuna triple vírica puede provocar autismo. *

Marca solo un óvalo.

	1	2	3	4	5	
Seguro que es falso	◯	◯	◯	◯	◯	Seguro que es verdadero

20. Los ejercicios que promueven la coordinación de las habilidades perceptivo-motoras pueden mejorar las destrezas en lecto-escritura. *

Marca solo un óvalo.

	1	2	3	4	5	
Seguro que es falso	◯	◯	◯	◯	◯	Seguro que es verdadero

21. La práctica repetida de algunos procesos mentales puede cambiar la forma y la estructura de algunas partes del cerebro. *

Marca solo un óvalo.

	1	2	3	4	5	
Seguro que es falso	◯	◯	◯	◯	◯	Seguro que es verdadero

22. Los niños tienen el cerebro más grande que las niñas. *

Marca solo un óvalo.

	1	2	3	4	5	
Seguro que es falso	◯	◯	◯	◯	◯	Seguro que es verdadero

23. Las diferencias entre los niños y las niñas son irrelevantes a la hora de enseñar a unos y a otros. *

Marca solo un óvalo.

	1	2	3	4	5	
Seguro que es falso						Seguro que es verdadero

24. Las personas aprenden mejor cuando reciben la información en su estilo de aprendizaje preferido (p.ej., auditivo, visual y cinestésico). *

Marca solo un óvalo.

	1	2	3	4	5	
Seguro que es falso						Seguro que es verdadero

25. Los hemisferios izquierdo y derecho del cerebro siempre trabajan juntos. *

Marca solo un óvalo.

	1	2	3	4	5	
Seguro que es falso						Seguro que es verdadero

26. Los deberes tienen mayores beneficios en alumnos de secundaria que en alumnos de primaria. *

Marca solo un óvalo.

	1	2	3	4	5	
Seguro que es falso						Seguro que es verdadero

27. Muchos problemas en lectura se pueden solucionar mediante ejercicios optométricos (p.ej., seguir los movimiento de una pelota con los ojos, contar letras línea por línea usando sólo los ojos). *

Marca solo un óvalo.

	1	2	3	4	5	
Seguro que es falso	◯	◯	◯	◯	◯	Seguro que es verdadero

28. El desarrollo normal del cerebro humano implica el nacimiento y muerte de células cerebrales. *

Marca solo un óvalo.

	1	2	3	4	5	
Seguro que es falso	◯	◯	◯	◯	◯	Seguro que es verdadero

29. Es más eficaz dar feedback incluyendo información sobre la respuesta correcta que incluyendo información sobre la respuesta incorrecta. *

Marca solo un óvalo.

	1	2	3	4	5	
Seguro que es falso	◯	◯	◯	◯	◯	Seguro que es verdadero

30. La información se almacena en una red de células distribuidas por todo el cerebro. *

Marca solo un óvalo.

	1	2	3	4	5	
Seguro que es falso	◯	◯	◯	◯	◯	Seguro que es verdadero

31. Solo usamos el 10% del cerebro. *

Marca solo un óvalo.

	1	2	3	4	5	
Seguro que es falso						Seguro que es verdadero

32. La producción de nuevas conexiones en el cerebro puede continuar hasta la vejez. *

Marca solo un óvalo.

	1	2	3	4	5	
Seguro que es falso						Seguro que es verdadero

33. Usamos el cerebro 24 horas al día. *

Marca solo un óvalo.

	1	2	3	4	5	
Seguro que es falso						Seguro que es verdadero

34. Se pueden reducir los problemas de aprendizaje y conducta mediante sesiones cortas de escucha de música modificada electrónicamente (p.ej., método Berard). *

Marca solo un óvalo.

	1	2	3	4	5	
Seguro que es falso						Seguro que es verdadero

35. Los entornos que son ricos en estímulos mejoran los cerebros de los niños y niñas preescolares. *

Marca solo un óvalo.

	1	2	3	4	5	
Seguro que es falso						Seguro que es verdadero

36. La instrucción directa (programación previa de los contenidos y de los criterios de evaluación, secuenciación de la dificultad, feedback, modelado, práctica guiada) conduce a mejores resultados que el aprendizaje por descubrimiento (p.ej., aprendizaje por proyectos, aprendizaje basado en problemas). *

Marca solo un óvalo.

	1	2	3	4	5	
Seguro que es falso						Seguro que es verdadero

37. Escuchar música de Mozart aumenta la inteligencia de los niños. *

Marca solo un óvalo.

	1	2	3	4	5	
Seguro que es falso						Seguro que es verdadero

38. Escribir letras en espejo NO es un síntoma de la dislexia. *

Marca solo un óvalo.

	1	2	3	4	5	
Seguro que es falso						Seguro que es verdadero

39. La proporción de niños diagnosticados con autismo es superior a la de las niñas. *

Marca solo un óvalo.

	1	2	3	4	5	
Seguro que es falso	◯	◯	◯	◯	◯	Seguro que es verdadero

¿Usaría o recomendaría el uso de alguna de las siguientes prácticas educativas?
Por favor, para cada cuestión marque la opción que mejor represente su valoración.

LEA ATENTAMENTE las opciones disponibles:

1. Seguro que NO
2. Muy improbable
3. Improbable
4. Probable
5. Muy probable
6. Seguro que SÍ

40. *

Selecciona todos los que correspondan.

☐ De acuerdo

41. Enseñar habilidades de autorregulación como facilitadoras del aprendizaje (p.ej., estrategias de organización y planificación). *

Marca solo un óvalo.

	1	2	3	4	5	6	
Seguro que NO	◯	◯	◯	◯	◯	◯	Seguro que SÍ

42. Métodos para promover el aprendizaje autónomo de los estudiantes a través de la web. *

Marca solo un óvalo.

	1	2	3	4	5	6	
Seguro que NO	◯	◯	◯	◯	◯	◯	Seguro que SÍ

43. Adaptar la enseñanza a los estilos de aprendizaje. *

Marca solo un óvalo.

	1	2	3	4	5	6	
Seguro que NO	◯	◯	◯	◯	◯	◯	Seguro que SÍ

44. Proveer al alumnado de feedback sobre qué ha hecho y cómo puede mejorar. *

Marca solo un óvalo.

	1	2	3	4	5	6	
Seguro que NO	◯	◯	◯	◯	◯	◯	Seguro que SÍ

45. Práctica distribuida de lo aprendido (espaciar en el tiempo los episodios de aprendizaje de un determinado contenido). *

Marca solo un óvalo.

	1	2	3	4	5	6	
Seguro que NO	◯	◯	◯	◯	◯	◯	Seguro que SÍ

46. Instrucción directa (programación previa de los contenidos y de los criterios de evaluación, secuenciación de la dificultad, feedback, modelado, práctica guiada). *

Marca solo un óvalo.

	1	2	3	4	5	6	
Seguro que NO	◯	◯	◯	◯	◯	◯	Seguro que SÍ

47. La práctica de determinados ejercicios físicos para restablecer o consolidar la lateralidad en casos de niños con lateralidad cruzada. *

Marca solo un óvalo.

	1	2	3	4	5	6	
Seguro que NO	◯	◯	◯	◯	◯	◯	Seguro que SÍ

48. Métodos de estimulación temprana para mejorar el cerebro de los niños y niñas preescolares (p.ej., Doman). *

Marca solo un óvalo.

	1	2	3	4	5	6	
Seguro que NO	◯	◯	◯	◯	◯	◯	Seguro que SÍ

49. Proveer al alumnado de ejemplos de problemas ya resueltos paso a paso. *

Marca solo un óvalo.

	1	2	3	4	5	6	
Seguro que NO	◯	◯	◯	◯	◯	◯	Seguro que SÍ

50. Evaluación frecuente de lo aprendido (como parte o no de la calificación final). *

Marca solo un óvalo.

	1	2	3	4	5	6	
Seguro que NO	◯	◯	◯	◯	◯	◯	Seguro que SÍ

51. Métodos para favorecer el uso del 100% del cerebro. *

Marca solo un óvalo.

	1	2	3	4	5	6	
Seguro que NO	◯	◯	◯	◯	◯	◯	Seguro que SÍ

52. Uso del método fonológico o sintético para la enseñanza inicial de la lectura (enseñar de forma explícita la asociación letra-sonido). *

Marca solo un óvalo.

	1	2	3	4	5	6	
Seguro que NO	◯	◯	◯	◯	◯	◯	Seguro que SÍ

53. Escuchar música de Mozart para aumentar la inteligencia. *

Marca solo un óvalo.

	1	2	3	4	5	6	
Seguro que NO	◯	◯	◯	◯	◯	◯	Seguro que SÍ

54. Ejercicios de coordinación para mejorar la integración de la función cerebral del hemisferio izquierdo y derecho. *

Marca solo un óvalo.

	1	2	3	4	5	6	
Seguro que NO	◯	◯	◯	◯	◯	◯	Seguro que SÍ

55. Método global para la enseñanza de la lectura (enseñar a leer palabras completas y no la asociación letra-sonido). *

Marca solo un óvalo.

	1	2	3	4	5	6	
Seguro que NO	◯	◯	◯	◯	◯	◯	Seguro que SÍ

56. Aprendizaje cooperativo (versus aprendizaje individual). *

Marca solo un óvalo.

	1	2	3	4	5	6	
Seguro que NO	◯	◯	◯	◯	◯	◯	Seguro que SÍ

57. Aceleración de curso en alumnos de altas capacidades. *

Marca solo un óvalo.

	1	2	3	4	5	6	
Seguro que NO	◯	◯	◯	◯	◯	◯	Seguro que SÍ

58. Ejercicios de coordinación de las habilidades perceptivo-motoras para favorecer la lecto-escritura. *

Marca solo un óvalo.

	1	2	3	4	5	6	
Seguro que NO	◯	◯	◯	◯	◯	◯	Seguro que SÍ

59. Indique si ha buscado información adicional sobre las siguientes afirmaciones EN LOS ÚLTIMOS 30 DÍAS. *

Selecciona todos los que correspondan.

- ☐ De acuerdo

60. Los entornos ricos en estímulos mejoran el cerebro de los niños y niñas. *

Marca solo un óvalo.

- ◯ No he buscado información.
- ◯ Sí he buscado información y va A FAVOR de esta afirmación.
- ◯ Sí he buscado información y va EN CONTRA de esta afirmación.
- ◯ No recuerdo haber buscado información.

61. Adaptar la enseñanza a los estilos de aprendizaje mejora el aprendizaje. *

Marca solo un óvalo.

- ◯ No he buscado información.
- ◯ Sí he buscado información y va A FAVOR de esta afirmación.
- ◯ Sí he buscado información y va EN CONTRA de esta afirmación.
- ◯ No recuerdo haber buscado información.

62. Los ejercicios perceptivo-motores mejoran la lecto-escritura. *

Marca solo un óvalo.

- No he buscado información.
- Sí he buscado información y va A FAVOR de esta afirmación.
- Sí he buscado información y va EN CONTRA de esta afirmación.
- No recuerdo haber buscado información.

63. Solo usamos el 10% del cerebro. *

Marca solo un óvalo.

- No he buscado información.
- Sí he buscado información y va A FAVOR de esta afirmación.
- Sí he buscado información y va EN CONTRA de esta afirmación.
- No recuerdo haber buscado información.

64. Las diferencias en el hemisferio dominante explican las diferencias entre estudiantes. *

Marca solo un óvalo.

- No he buscado información.
- Sí he buscado información y va A FAVOR de esta afirmación.
- Sí he buscado información y va EN CONTRA de esta afirmación.
- No recuerdo haber buscado información.

65. Las nuevas generaciones tienen habilidades tecnológicas sofisticadas para aprender de la web. *

Marca solo un óvalo.

- No he buscado información.
- Sí he buscado información y va A FAVOR de esta afirmación.
- Sí he buscado información y va EN CONTRA de esta afirmación.
- No recuerdo haber buscado información.

66. Escuchar música Mozart aumenta la inteligencia. *

Marca solo un óvalo.

- No he buscado información.
- Sí he buscado información y va A FAVOR de esta afirmación.
- Sí he buscado información y va EN CONTRA de esta afirmación.
- No recuerdo haber buscado información.

67. La lateralidad cruzada está asociada a las dificultades de aprendizaje. *

Marca solo un óvalo.

- No he buscado información.
- Sí he buscado información y va A FAVOR de esta afirmación.
- Sí he buscado información y va EN CONTRA de esta afirmación.
- No recuerdo haber buscado información.

68. El método global es el más eficaz para enseñar a leer. *

Marca solo un óvalo.

- No he buscado información.
- Sí he buscado información y va A FAVOR de esta afirmación.
- Sí he buscado información y va EN CONTRA de esta afirmación.
- No recuerdo haber buscado información.

69. Indique si ha recibido información sobre alguna de estas afirmaciones A LO LARGO DE SUS ESTUDIOS DE MAGISTERIO o FORMACIÓN CONTINUA. *

Selecciona todos los que correspondan.

- [ ] De acuerdo

70. Los entornos ricos en estímulos mejoran el cerebro de los niños y niñas. *

Marca solo un óvalo.

- No he recibido información.
- Sí he recibido información y va A FAVOR de esta afirmación.
- Sí he recibido información y va EN CONTRA de esta afirmación.
- No recuerdo haber recibido información.

71. Adaptar la enseñanza a los estilos de aprendizaje mejora el aprendizaje. *

Marca solo un óvalo.

- No he recibido información.
- Sí he recibido información y va A FAVOR de esta afirmación.
- Sí he recibido información y va EN CONTRA de esta afirmación.
- No recuerdo haber recibido información.

72. Los ejercicios perceptivo-motores mejoran la lecto-escritura. *

Marca solo un óvalo.

- No he recibido información.
- Sí he recibido información y va A FAVOR de esta afirmación.
- Sí he recibido información y va EN CONTRA de esta afirmación.
- No recuerdo haber recibido información.

73. Solo usamos el 10% del cerebro. *

Marca solo un óvalo.

- No he recibido información.
- Sí he recibido información y va A FAVOR de esta afirmación.
- Sí he recibido información y va EN CONTRA de esta afirmación.
- No recuerdo haber recibido información.

74. Las diferencias en el hemisferio dominante explican las diferencias entre estudiantes. *

Marca solo un óvalo.

- No he recibido información.
- Sí he recibido información y va A FAVOR de esta afirmación.
- Sí he recibido información y va EN CONTRA de esta afirmación.
- No recuerdo haber recibido información.

75. Las nuevas generaciones tienen habilidades tecnológicas sofisticadas para aprender de la web. *

Marca solo un óvalo.

- No he recibido información.
- Sí he recibido información y va A FAVOR de esta afirmación.
- Sí he recibido información y va EN CONTRA de esta afirmación.
- No recuerdo haber recibido información.

76. Escuchar música Mozart aumenta la inteligencia. *

Marca solo un óvalo.

- No he recibido información.
- Sí he recibido información y va A FAVOR de esta afirmación.
- Sí he recibido información y va EN CONTRA de esta afirmación.
- No recuerdo haber recibido información.

77. La lateralidad cruzada está asociada a las dificultades de aprendizaje. *

Marca solo un óvalo.

- No he recibido información.
- Sí he recibido información y va A FAVOR de esta afirmación.
- Sí he recibido información y va EN CONTRA de esta afirmación.
- No recuerdo haber recibido información.

78. El método global es el más eficaz para enseñar a leer. *

Marca solo un óvalo.

- No he recibido información.
- Sí he recibido información y va A FAVOR de esta afirmación.
- Sí he recibido información y va EN CONTRA de esta afirmación.
- No recuerdo haber recibido información.

Este contenido no ha sido creado ni aprobado por Google.

Google Formularios
